# A multi-trait Bayesian method for mapping QTL and genomic prediction

**DOI:** 10.1186/s12711-018-0377-y

**Published:** 2018-03-24

**Authors:** Kathryn E. Kemper, Philip J. Bowman, Benjamin J. Hayes, Peter M. Visscher, Michael E. Goddard

**Affiliations:** 1 Faculty of Veterinary and Agricultural Sciences, University of Melbourne, Parkville, 3052 Australia; 2 Institute of Molecular Biosciences, University of Queensland, St. Lucia, 4072 Australia; 3 Agriculture Victoria, AgriBio, Centre for AgriBioscience, Bundoora, VIC 3083 Australia; 4 School of Applied Systems Biology, La Trobe University, Bundoora, VIC 3083 Australia; 5 Queensland Agriculture and Food Alliance, University of Queensland, St. Lucia, 4072 Australia; 6 Dairy Futures Co-operative Research Centre, Bundoora, 3083 Australia; 7 Queensland Brain Institute, University of Queensland, St. Lucia, 4072 Australia

## Abstract

**Background:**

Genomic prediction and quantitative trait loci (QTL) mapping typically analyze one trait at a time but this may ignore the possibility that one polymorphism affects multiple traits. The aim of this study was to develop a multivariate Bayesian approach that could be used for simultaneously elucidating genetic architecture, QTL mapping, and genomic prediction. Our approach uses information from multiple traits to divide markers into ‘unassociated’ (no association with any trait) and ‘associated’ (associated with one or more traits). The effect of associated markers is estimated independently for each trait to avoid the assumption that QTL effects follow a multi-variate normal distribution.

**Results:**

Using simulated data, our multivariate method (BayesMV) detected a larger number of true QTL (with a posterior probability > 0.9) and increased the accuracy of genomic prediction compared to an equivalent univariate method (BayesR). With real data, accuracies of genomic prediction in validation sets for milk yield traits with high-density genotypes were approximately equal to those from equivalent single-trait methods. BayesMV tended to select a similar number of single nucleotide polymorphisms (SNPs) per trait for genomic prediction compared to BayesR (i.e. those with non-zero effects), but BayesR selected different sets of SNPs for each trait, whereas BayesMV selected a common set of SNPs across traits. Despite these two dramatically different estimates of genetic architecture (i.e. different SNPs affecting each trait vs. pleiotropic SNPs), both models indicated that 3000 to 4000 SNPs are associated with a trait. The BayesMV approach may be advantageous when the aim is to develop a low-density SNP chip that works well for a number of traits. SNPs for milk yield traits identified by BayesMV and BayesR were also found to be associated with detailed milk composition.

**Conclusions:**

The BayesMV method simultaneously estimates the proportion of SNPs that are associated with a combination of traits. When applied to milk production traits, most of the identified SNPs were associated with all three traits (milk, fat and protein yield). BayesMV aims at exploiting pleiotropic QTL and selects a small number of SNPs that could be used to predict multiple traits.

**Electronic supplementary material:**

The online version of this article (10.1186/s12711-018-0377-y) contains supplementary material, which is available to authorized users.

## Background

Understanding the genetics of quantitative or complex traits has been revolutionized by the availability of dense panels of single nucleotide polymorphisms (SNPs) that cover the genome. Data on SNP genotypes combined with phenotypic measurements have been used for three purposes: to study the genetic architecture of quantitative traits, to map regions of the genome that cause variation in these traits (quantitative trait loci or QTL) and to predict the genetic or breeding values of individuals for quantitative traits. Although different statistical methods are commonly used for these three purposes, we have argued that a non-linear Bayesian method that fits all SNPs simultaneously can be used for all three aims [1, 2]. For example, BayesR makes across-breed predictions of breeding values and maps QTL more accurately than genomic best linear unbiased prediction (GBLUP) [1].

QTL often affect more than one trait [3, 4] but most methods that are applied to analyze SNP data use only one trait at a time. Multivariate analyses have been found to increase power to detect and map QTL [5] and increase the accuracy of estimated breeding values (EBV) [6]. For instance, QTL mapping is frequently performed in genome-wide association studies (GWAS) by single-SNP regression, in which the effect of one SNP at a time on the trait is tested. Multiple-trait versions of single-SNP regression have been implemented in various studies e.g. [7–9]. Multi-trait EBV are frequently calculated from pedigree data or from SNP genotypes using BLUP, and sometimes have a higher accuracy compared to single-trait EBV [10, 11]. In dairy cattle, multi-trait genomic prediction models using multi-breed populations (i.e. where a trait measured in different breeds is treated as multiple traits) have been attempted in several studies to account for between-breed differences in QTL effects but with limited success [12–16]. The focus of our paper is the development of a Bayesian multi-trait genomic prediction method for multiple different traits.

Jia and Jannink [17] and Calus and Veerkamp [18] have described non-linear Bayesian methods for the analysis of high-density SNP data on multiple traits. However, these methods assume that the effects of a QTL on different traits are drawn from a multivariate normal distribution with the same correlation for all QTL, which may be incorrect. For instance, an allele of the gene *DGAT1* (*diacylglycerol O*-*acyltransferase 1*) increases milk yield but decreases milk fat yield in spite of a positive overall genetic correlation between milk and fat yields [19]. Kemper et al. [1] reported numerous similar cases where the pattern of effects of QTL on traits varied, while the overall genetic correlation between milk traits was positive. Some QTL may also affect two traits although there is a weak genetic correlation between the traits. Thus, the assumption of multivariate normality may be too strong.

The aim of our study was to develop a multivariate version of BayesR that uses multiple trait data to decide which SNPs should be included in the model but allows flexibility in the estimation of the effect of the selected SNPs on each of the traits analyzed by estimating the effect of selected SNPs independently for each trait. The hypothesis under investigation is that multi-trait information will improve accuracies of genomic prediction. We illustrate the method by applying it to simulated data for QTL mapping and genomic prediction, and to real data on milk, fat and protein yields from dairy cattle.

## Methods

### Real data

The available dairy cattle dataset had over 16,000 records on Holstein and Jersey cattle from a previous study [1]. The reference population consisted of up to 11,527 Holstein and 4687 Jersey animals, while the validation dataset (used to evaluate the accuracy of genomic predictions) consisted of phenotype records for up to 262 Holstein bulls, 105 Jersey bulls, and 361 Australian Red (bull and cow) cattle (Table 1). Australian red cattle were never included in the reference population, so validation for these animals represents across-breed prediction. Australian red cattle are more closely related to Holstein than to Jersey cattle, as detailed in “Appendix 1”.

**Table 1:** Number of records in the reference and validation datasets for Holstein, Jersey and Australian Red dairy cattle

Breed	Traits	Total records	Reference		Validation		
			Bulls	Cows	YOB	Bulls	Cows
Holstein	FY, MY, PY	11,789	3049	8478	2005	262	–
Jersey	FY, MY, PY	4793	770	3917	2005	105	–
Australian Red*	FY, MY, PY	361	–	–	–	114	247

Year-of-birth (YOB) for the animals included in the validation datasets is also provided

FY = fat yield (kg/lac), MY = milk yield (L/lac); PY = protein yield (kg/lac)

*Australian Red animals were only used for validation and never included in the reference population

Phenotypes are three yield traits (fat, milk and protein yield, i.e. FY, MY and PY, respectively) supplied as either daughter-trait deviations (for males) or trait-deviations (for females) from the Australian Dairy Herd Improvement Scheme [1]. Heterogeneous error variances in the phenotypes were accounted for by using a weighted analysis, using the weighting procedure outlined by Garrick et al. [20] and described in detail for this dataset by Kemper et al. [1]. Trait heritabilities and phenotypic and genetic correlations between traits were estimated using the relationship matrix constructed from pedigree data using ASReml [21]. All animals had real or imputed genotypes for 632,002 SNPs from the bovine HD array [1, 22]. Full details of the imputation and quality control procedures for genotypes are described in [22] but they include removal of SNPs with very poor imputation accuracy and SNPs with low minor allele frequency (less than 10 copies in the dataset).

GWAS summary statistics (i.e. allele name, allele effect and standard deviation) were available from a previous study [23] which used a subset of Holstein cows (N = 444) from this dataset. These statistics were for detailed milk phenotypes and we used them to verify QTL that were identified using the Bayesian approaches.

### Simulated data

Additive QTL were simulated on real dairy cattle genotypes for a single chromosome to illustrate the impact of the multi-trait method on (1) the accuracy of genomic predictions, (2) the ability to elucidate genetic architecture and (3) the power of QTL mapping. In the simulation, genotypes consisted of 12,745 SNPs from *Bos taurus* autosome 29 (BTA29) for 3049 Holstein animals from the larger reference dataset described above. Simulations involved two traits with 10 QTL per trait, two scenarios with a different number of pleiotropic QTL and 20 replicates for each scenario. In scenario 1, there were no pleiotropic QTL (pQTL = 0) and 20 SNPs were randomly selected as QTL with effects on either trait 1 or trait 2, separately for each replicate. In scenario 2, all QTL had pleiotropic effects (pQTL = 1) and thus, 10 SNPs were randomly selected for each replicate to have effects on both traits. All SNPs chosen as QTL had a minor allele frequency higher than 0.01. Additive QTL effects explained 0.01 of the phenotypic variance ($\sigma_P^2$) and were calculated for each chosen SNP as $\sqrt {0.01\sigma_P^2/2a\left({1 - a} \right)}$, where *a* is the allele frequency of the SNP, and were randomly allocated to have either positive or negative effects on the trait. Thus, the heritability of the traits was 0.10. The error co-variance between traits was zero.

### Statistical analysis

#### Construction of traits with independent errors

Method BayesMV (described later) requires uncorrelated residual errors. Thus, we used a principal component analysis to form linear combinations of the three milk yield traits to create new traits with zero error covariance. A principal component decomposition was conducted on the error correlation matrix (**K**, a *t* × *t* matrix where *t* = number of traits) from the pedigree-based multivariate analysis in ASReml [21] [see Additional file 1: Table S1]. Then ${\bf{K}} = {\bf{B}}\varvec{\varLambda} {\bf{B}}^\prime$, where $\varvec{\varLambda}$ is a diagonal matrix of eigenvalues and ${\mathbf{B}}$ is a *t* × *t* matrix of eigenvectors. The linear combinations (${\mathbf{LC}}$) of traits were constructed by ${\mathbf{LC}} = {\mathbf{B}}^{{\prime }} {\mathbf{T}}^{ - 1} {\mathbf{y}}$, where ${\mathbf{T}}$ is a diagonal matrix of error standard deviations and ${\mathbf{y}}$ is the vector of phenotypes. The genetic variance of the linear combinations is ${\mathbf{B}}{\prime }{\mathbf{T}}^{ - 1} {\mathbf{GT}}^{ - 1} {\mathbf{B}}$, where ${\mathbf{G}}$ is the genetic variance–covariance matrix. Estimated breeding values for the original traits were constructed for an individual as ${\mathbf{T}} {\mathbf{B}} {\mathbf{LC}}_{{{\mathbf{GEBV}}}}$, where ${\mathbf{LC}}_{{{\mathbf{GEBV}}}}$ is the vector of EBV for the linear combination traits.

#### Statistical model

The model fitted to the data for both BayesR and BayesMV had the general form ${\mathbf{y}} = {\mathbf{Xb}} + {\mathbf{Za}} + {\mathbf{Wv}} + {\mathbf{e}}$, where ${\mathbf{y}}$ is the vector of phenotypes (i.e. for the linear combination of traits), ${\mathbf{b}}$ is a vector of fixed effects, ${\mathbf{a}}$ is a vector of polygenic breeding values not explained by the SNPs [distributed as $N\left( {0,{\mathbf{A}}\sigma_{a}^{2} } \right)$, where ${\mathbf{A}}$ is the numerator relationship matrix (from pedigree) and $\sigma_{a}^{2}$ is the additive genetic variance not explained by the SNP], ${\mathbf{v}}$ is a vector of SNP effects assumed normally distributed [$N\left( {0,\sigma_{k}^{2} } \right)$, with $k = 1, 2, 3\,\, {\text{or}}\,\, 4$: $\sigma_{1}^{2} = 0$, $\sigma_{2}^{2} = 0.0001\sigma_{{a^{*} }}^{2}$, $\sigma_{3}^{2} = 0.001\sigma_{{a^{*} }}^{2}$ and $\sigma_{4}^{2} = 0.01\sigma_{{a^{*} }}^{2}$, where $\sigma_{{a^{*} }}^{2}$ is the additive genetic variance estimated from pedigree], ${\mathbf{W}}$ is a matrix of standardized SNP genotypes (as defined in “Appendix 2”), and ${\mathbf{e}}$ is a vector of residual errors [distributed as $N\left( {0,{\mathbf{R}}} \right)$, where ${\mathbf{R}}$ is the error covariance matrix, ${\mathbf{R}} = {\mathbf{E}}\sigma_{e}^{2}$ and ${\mathbf{E}}^{ - 1}$ is a diagonal matrix of error weights]. We fitted both the univariate (BayesR) and multivariate (BayesMV) models to the linear combinations. A full description of the univariate BayesR used is in [1], while method BayesMV is described in the next section. BayesMV differs from BayesR in that a proportion of the SNPs ($p$) are defined as ‘unassociated’ and are assumed to have no effect on any trait. BayesR is equivalent to assuming that $p = 0$, so all SNPs are associated and effects are estimated separately for each trait.

#### BayesMV

In the BayesMV method, the traits are analyzed simultaneously. It is assumed that the residuals (${\mathbf{e}}$) and polygenic effects (${\mathbf{a}}$) are independent between traits and the only connection between traits is the model for the SNP effects (${\mathbf{v}}$). SNPs are either ‘associated’ with prior probability $1 - p$ or ‘unassociated’ with prior probability $p$. If a SNP is unassociated, it has no effect on any trait. If a SNP is associated, the effects on each trait are assumed to be independent and drawn from a mixture of normal distributions, $N\left( {0,\sigma_{k}^{2} } \right)$, with $k = 1, 2, 3 {\text{or }}4$: $\sigma_{1}^{2} = 0$, $\sigma_{2}^{2} = 0.0001\sigma_{{a^{*} }}^{2}$, $\sigma_{3}^{2} = 0.001\sigma_{{a^{*} }}^{2}$ and $\sigma_{4}^{2} = 0.01\sigma_{{a^{*} }}^{2}$, where $\sigma_{{a^{*} }}^{2}$ is the additive genetic variance estimated from pedigree [22]. Thus, the probability that the effects of a SNP are drawn from each distribution depends on $p$ (the probability that the SNP is unassociated) and $q_{j,k}$ (the mixing proportion of distribution $k$ for trait $j$ conditional on the SNP being associated). For example, consider two traits and distribution 1 for trait 1 and distribution 2 for trait 2. The probability that the effect of the SNP belongs to distributions $k1$ and $k2$ is equal to $p + \left( {1 - p} \right)q_{1,k1} q_{2,k2}$ when $k1 = k2 = 1$ and equal to $\left( {1 - p} \right)q_{1,k1} q_{2,k2}$ otherwise. Full details of the model and the Gibbs sampler used to implement the model are in “Appendix 2”. Reported effects are posterior means of samples from the Gibbs sampler with at least 30,000 iterations, with 20,000 iterations discarded as burn-in. Final results are the mean of five replicate chains.

#### Accuracy and bias of genomic predictions

The accuracy of the genomic predictions was assessed in the validation population of young Holstein (N = 262), Jersey (N = 105) bulls, and Australian Red animals (N = 361) (Table 1). In simulations, accuracies were calculated as r(TBV, GEBV), where TBV is the true breeding value and GEBV is the breeding value predicted using SNP genotypes. In the real data, the accuracy of genomic predictions was the correlation of predicted breeding values with daughter-deviations (for bulls) or phenotypes (for cows) in the validation dataset, i.e. $r\left( {{\hat{\mathbf{y}}},{\mathbf{y}}} \right)$ where ${\mathbf{y}}$ is a vector of phenotypes for the validation population and ${\hat{\mathbf{y}}}$ is a vector of GEBV for the corresponding animals in ${\mathbf{y}}$. The bias of the predictions was assessed as the regression slope $b\left( {{\mathbf{y}},{\hat{\mathbf{y}}}} \right)$, where an unbiased (ideal) prediction has a regression slope of 1. Accuracy and bias for Australian Reds were computed as the average results for bulls and cows.

### Association analyses

A multi-trait single-SNP regression association analysis method [4] was used in the simulated data for QTL mapping for comparison to the Bayesian approaches. GWAS association summary statistics from a previous study [23] were used to verify QTL regions identified by using BayesMV and BayesR. This data consisted of the allele effect and standard deviation for concentration of lactose, minerals (calcium, potassium, magnesium, sodium, phosphorus, sulfate and zinc; mg/kg), and proteins (lactoperoxidase, lactoferrin, immunoglobulinG, alpha-lactalbumin, beta-lactoglobulin, kappa-casein, alpha-S1-casein and beta-casein; mg/g) in milk. Briefly, the analysis was for up to 444 cows for which trait records had been corrected for non-genetic effects, such as herd-year-season and stage of lactation, and a mixed linear model was used to detect associations between these traits and 609,563 autosomal SNPs with a minor allele frequency higher than 0.01. The –mlma-loco option using GCTA [24] was used which fits a genomic relationship matrix to account for population structure, where SNPs from the chromosome under test are excluded from the relationship matrix and the tested SNPs are fitted one-at-a-time as fixed effects.

## Results

### Simulated data

#### Genetic architecture

In the simulated dataset, there were 20 and 10 QTL with non-zero effects on one or both traits for scenarios 1 and 2, respectively, and all QTL had a variance of 0.01 $\sigma_{P}^{2}$. Table 2 shows the posterior number of SNPs from joint distributions for traits 1 and 2, for BayesR and BayesMV (where the joint distribution for BayesR was calculated as the product of the posterior probabilities for each trait). For example, when pQTL = 0, BayesMV estimated that 25 SNPs were only associated with trait 1, 23 were only associated with trait 2 and 41 SNPs were associated with both traits. Considering only trait 1, the number of SNPs estimated to have no effect (i.e. to have an effect sampled from distribution 1) was equal to 12,569 (12,396 + 173) for BayesR and 12,680 (12,643 + 14 + 23) for BayesMV. BayesR estimated similar distributions for both scenarios; i.e. hundreds of SNPs were associated with either trait 1 or trait 2, and two to three SNPs were associated with both traits. BayesMV estimated the true mixing proportions more accurately especially when the same SNPs affected both traits in the simulated data (pQTL = 1). When the same SNPs affected both traits (pQTL = 1), BayesMV estimated that most of the 17 SNPs classified as associated with both traits (i.e. they were sampled from non-zero distributions) and the remaining two SNPs were associated with either trait 1 or trait 2. However, when different SNPs were simulated to affect the two traits, BayesMV estimated an inflated number of SNPs to be associated with both traits (i.e. 41 when no SNPs were simulated to affect both traits). Both BayesR and BayesMV fitted more SNPs in the model than the number of QTL simulated, probably because they fit multiple SNPs for a given QTL, each with a small variance, instead of only 10 SNPs with a large variance.

**Table 2:** Posterior mean number of SNPs allocated to each component of the mixture distribution for the two simulated traits when analyzed with BayesR or BayesMV

pQTL^a^	Distribution^b^	Simulated^c^	BayesR^d^	BayesMV
0.0	Unassociated	12,725	–	12,643
	(trait1)_1__(trait2)_1_	0	12,396	14
	(trait1)_2–4__(trait2)_1_	10	174	25
	(trait1)_1__(trait2)_2–4_	10	173	23
	(trait1)_2–4__(trait2)_2–4_	0	2	41
1.0	Unassociated	12,735	–	12,728
	(trait1)_1__(trait2)_1_	0	12,435	0
	(trait1)_2–4__(trait2)_1_	0	148	1
	(trait1)_1__(trait2)_2–4_	0	159	1
	(trait1)_2–4__(trait2)_2–4_	10	3	15

^a^QTL were independent (no pleiotropic QTL, pQTL = 0) or completely pleiotropic (pQTL = 1.0)

^b^Subscripts indicate distributions 1 to 4, where distributions 1 to 4 explain 0, 0.0001, 0.001 or 0.01 σ_P_^2^, respectively

^c^The number of simulated QTL is also provided

^d^Joint probabilities are the product of posterior probabilities (*p* and *q*)

#### QTL mapping

**Fig. 1:**
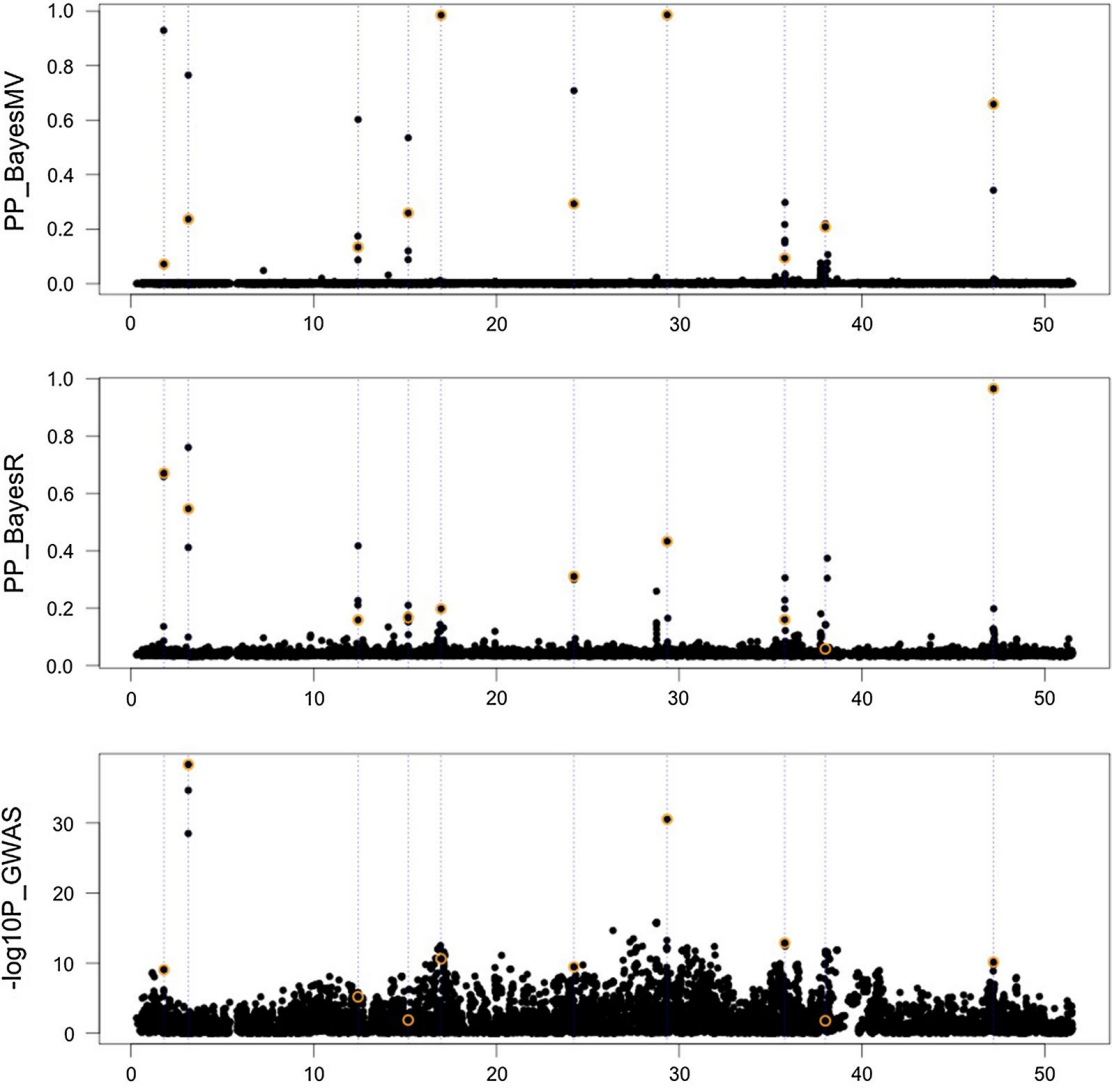
QTL mapping in simulated data where all QTL are pleiotropic. The mean posterior probabilities (PP) of SNPs having a non-zero effect for any trait for multivariate (BayesMV, top) and univariate (BayesR, middle) methods with the –log10(*P* value) for the multi-trait single-SNP genome-wide association study (GWAS, bottom) are shown. Results for simulated QTL are highlighted in orange and their position marked with dashed vertical lines

Figure 1 illustrates the results from the BayesMV, BayesR, and (the multi-trait) single-SNP regression analyses for a single replicate dataset where all QTL were pleiotropic (pQTL = 1). The mean posterior probability (PP) that a SNP had a non-zero effect on any trait was the criterion for mapping QTL. This quantity is estimated directly in BayesMV and was calculated as 1 – (probability of being in the zero distribution for all traits) in BayesR. In the simulated data, the QTL were among the SNPs analyzed, so the ideal outcome is that these SNPs have a high PP and all others have a low PP. As shown in Fig. 1, the QTL were mapped quite accurately by BayesMV, since all the high PP SNPs were either a causative SNP or very close to one. BayesMV tended to have zero or close to zero PP for the remaining SNPs. Compared to BayesMV, BayesR showed more SNPs with a low PP. It is very difficult from the single-SNP regression GWAS to determine the location and possible number of QTL in the simulated dataset. All methods sometimes assigned the highest PP or − log10(P) to non-causal SNPs, demonstrating the influence of linkage disequilibrium (LD) and other nearby QTL on the association statistics in the cattle genotypes.

Results over the 20 replicate datasets are summarized in Figure S1 [see Additional file 2: Figure S1]. In scenarios where all QTL were pleiotropic (pQTL = 1.0), BayesMV had greater power to detect QTL (i.e. where power = number of detected causative SNPs/total number of causative SNPs) when the PP was high (PP > 0.9). This was achieved with approximately the same false-discovery rate (FDR), (i.e. number of detected unassociated SNPs/total number of detected SNPs) as BayesR. In simulations with no pleiotropy, BayesMV had similar power but a higher FDR than BayesR. This was likely caused by SNPs that influenced trait 2 being sampled more frequently from the non-zero distributions for trait 1 (i.e. joint probabilities from BayesMV indicated that there were 41 SNPs that had non-zero effects for both traits but no QTL were simulated to affect both traits).

#### Genomic prediction

The accuracy of predictions from the simulated data were high (~ 0.90) when validated in a population similar to the reference dataset (Holstein) but lower when used for across-breed prediction (validated in Jersey, Table 3). Accuracies were much higher than what is typically observed in real data but this is as expected when causative QTL are included in the analyzed set of SNPs, and when the sizes of QTL effects are relatively large (0.01 $\sigma_{P}^{2}$). In simulations without pleiotropic QTL (pQTL = 0), BayesR and BayesMV achieved very similar accuracies of prediction, both for the Holstein and the Jersey validation datasets (Table 3). However, when all QTL were pleiotropic (pQTL = 1.0), BayesMV had significantly higher prediction accuracy than BayesR by on average 0.08 for Holstein validation and 0.14 for Jersey validation.

**Table 3:** Accuracy of genomic predictions for the analysis of the two simulated traits with BayesR and BayesMV for two pleiotropy scenarios

Method	Pleiotropy^a^	Trait	Holstein		Jersey	
			Accuracy	SE	Accuracy	SE
BayesR	pQTL = 0	1	0.88	0.07	0.78	0.15
		2	0.88	0.04	0.82	0.10
	pQTL = 1.0	1	0.89	0.05	0.77	0.15
		2	0.89	0.04	0.80	0.14
BayesMV	pQTL = 0	1	0.89	0.06	0.80	0.15
		2	0.90	0.04	0.84	0.11
	pQTL = 1.0	1	0.97	0.02	0.96	0.04
		2	0.97	0.01	0.96	0.04

*SE* standard error (across replicates)

^a^QTL were independent (no pleiotropy, pQTL = 0) or completely pleiotropic (pQTL = 1.0) and accuracies are for validation within-(Holstein) or across (Jersey) breeds

### Real data

#### Linear decomposition of traits

Milk yield traits had moderate heritabilities (h^2^ ~ 0.5) and moderate-high genetic correlations between them (0.5 to 0.8) [see Additional file 1: Table S1]. The eigenvector coefficients for the milk production traits showed that the first linear combination (LC1) was positively correlated to all three yield traits, the second linear combination (LC2) was primarily fat yield corrected for milk and protein yield, and the third linear combination (LC3) was protein yield corrected for milk yield (Table 4). Heritability estimates for the linear combination of traits were moderate to high (0.45 to 0.88, Table 4).

**Table 4:** Eigenvectors applied to each trait to construct linear combinations (LC1, 2 and 3) with zero error co-variance for the milk production traits

	LC1	LC2	LC3
Fat yield	0.55	0.83	− 0.01
Milk yield	0.59	− 0.39	− 0.70
Protein yield	0.59	− 0.39	0.71
Heritability (h^2^)^a^	0.45	0.73	0.88

^a^Estimated heritability of the linear combinations

#### Genetic architecture

The distribution of SNPs across the four distributions was relatively consistent between BayesR and BayesMV (Table 5), i.e., both methods found that 3000 to 4000 SNPs had non-zero effects for each trait. These findings were also consistent with the analysis of the original milk yield traits [see Additional file 3: Table S2]. The difference between the methods was that BayesMV explained all three traits by the same SNPs. For example, only 4092 associated SNPs were identified in the combined Holstein/Jersey reference set, most of which had effects for all three traits. For instance, joint probabilities identified only one SNP in the associated class that had a zero effect for all three traits, 79 SNPs with effects for only one trait, 949 SNPs with effects for two traits, and 3062 SNPs with effects for all three traits. In contrast, for BayesR, although the number of SNPs with non-zero effects per trait was similar to that observed for BayesMV, almost 11,500 SNPs had effects for at least one trait and only one SNP had effects for all three traits. Both models estimated that the Holstein or Jersey reference populations each had fewer associated SNPs (i.e. with non-zero effects) than the combined breed reference population. This is as expected if some QTL segregate in one breed only.

**Table 5:** Posterior mean number of SNPs^a^ in each distribution for milk production traits from BayesMV or BayesR

Reference	Distribution^b,c^	BayesR	BayesMV
Hol_Jer	Unassociated	–	627,911
	LC1_1__LC2_1__LC3_1_	620,515	1
	LC1_1__LC2_1__LC3_2–4_	3504	11
	LC1_1__LC2_2–4__LC3_1_	2994	4
	LC1_2–4__LC2_1__LC3_1_	4913	64
	LC1_1__LC2_2–4__LC3_2–4_	21	47
	LC1_2–4__LC2_1__LC3_2–4_	29	685
	LC1_2–4__LC2_2–4__LC3_1_	25	218
	LC1_2–4__LC2_2–4__LC3_2–4_	1	3062
Holstein	Unassociated	–	628,451
	LC1_1__LC2_1__LC3_1_	621,268	0
	LC1_1__LC2_1__LC3_2–4_	2817	2
	LC1_1__LC2_2–4__LC3_1_	3110	4
	LC1_2–4__LC2_1__LC3_1_	4743	12
	LC1_1__LC2_2–4__LC3_2–4_	17	50
	LC1_2–4__LC2_1__LC3_2–4_	22	124
	LC1_2–4__LC2_2–4__LC3_1_	25	234
	LC1_2–4__LC2_2–4__LC3_2–4_	0	3124
Jersey	Unassociated	–	630,779
	LC1_1__LC2_1__LC3_1_	624,314	0
	LC1_1__LC2_1__LC3_2–4_	1957	3
	LC1_1__LC2_2–4__LC3_1_	1366	3
	LC1_2–4__LC2_1__LC3_1_	4335	1
	LC1_1__LC2_2–4__LC3_2–4_	6	101
	LC1_2–4__LC2_1__LC3_2–4_	14	28
	LC1_2–4__LC2_2–4__LC3_1_	10	30
	LC1_2–4__LC2_2–4__LC3_2–4_	0	1057

^a^The posterior mean number of unassociated SNPs from BayesMV is shown with the joint probability of a non-zero effect on one or more traits. Joint probabilities are the product of posterior probabilities (*p* and *q*)

^b^Traits are three linear combinations (LC1, LC2, LC3) of fat, milk and protein yield

^c^Subscripts indicate distributions 1 to 4, each explaining 0, 0.0001, 0.001 or 0.01 of the genetic variance

#### QTL mapping

QTL mapping by BayesMV was assessed in the real data by investigating the top 100 SNPs with the highest PP for inclusion in the model [see Additional file 4: Table S3]. These 100 SNPs were grouped into four types of QTL based on their pattern of effects on milk, fat and protein yield, where the effect of a SNP for a trait was transformed from the LC as ${\mathbf{T}} {\mathbf{B}} {\mathbf{LC}}_{{{\mathbf{SNP}}}}$, with ${\mathbf{LC}}_{{{\mathbf{SNP}}}}$ being the vector of SNP effects for the linear combinations and ${\mathbf{B}}$ the matrix of eigenvectors from Table 4. The largest group of SNPs corresponded to those that had opposite effects on fat yield versus milk volume and protein yield. This group included several previously mapped loci such as *DGAT1* [19], *GPAT4* (*glycerol*-*3*-*phosphate acyltransferase 4*) [25] and *MGST1* (*microsomal glutathione S*-*transferase 1*) [26]. The second largest group of SNPs corresponded to those where an allele increased milk volume and milk solids (fat and protein yields). In some cases, two SNPs that were less than 100 Mbp apart showed a high PP but different patterns of effects, potentially indicating two or more QTL (i.e. BTA3 at about 15.5 Mbp). We chose two regions on chromosomes 11 and 19 for further investigation, where SNPs with a high PP from the BayesMV analysis also overlapped with SNPs associated with either lactoglobulin or potassium concentration [see Additional file 5: Table S4].

**Fig. 2:**
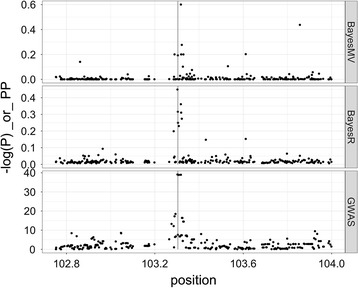
Mean posterior probability and associations with beta-lactoglobulin concentration on bovine chromosome 11. The posterior probability (PP) for a SNP being associated with any trait from BayesMV and BayesR and the single-SNP association analysis results are shown. The *PAEP* coding region (formally *LGB*, *lactoglobulin beta*) is highlighted in grey

The mean PP from BayesMV and BayesR on chromosome 11 is shown in Fig. 2. The SNP identified by BayesMV is located downstream of *PAEP* (*progestagen associated endometrial protein*; formally known as *LGB* or *lactoglobulin beta*), while the SNP identified by BayesR is within the coding region of *PAEP*. The figure also shows the GWAS results for beta-lactoglobulin concentration, for which the SNPs identified by both BayesMV (BovineHD1100030073) and BayesR (BovineHD1100030066) were among those that were most highly associated with beta-lactoglobulin concentration (P < 1 × 10^−39^; effect ≈ 0.68 mg/g). Although a QTL near *LGB* is known to affect milk volume, fat and protein yields [27], identification of the causal mutation for this QTL has been difficult due to strong LD in the region. The effect of the SNP that was identified by BayesMV had opposite effects on fat yield versus milk and protein yield.

The second QTL investigated was a novel region on chromosome 19, near the *KCNJ2* (*potassium voltage*-*gated channel subfamily J member 2*) gene [see Additional file 6: Figure S2]. The BovineHD1900017548 SNP was the most significant variant in the region for potassium concentration (P = 1.21 × 10^−7^; effect = − 62.87 mg/kg) and had a high PP with both BayesR and BayesMV. This SNP had opposite effects on protein yield versus milk and fat yield. KCNJ2 is potassium transporter that tends to transport potassium into (rather than out of) the cell (NCBI GeneID: 3759 [28]).

#### Genomic prediction

Genomic prediction accuracies for milk yield traits were moderate to high for breeds that were included in the reference population (~ 0.65 for FY, 0.62 for MY, and 0.58 for PY in Holstein; 0.57 for FY, 0.69 for MY, and 0.71 for PY in Jersey) but relatively low for Australian Reds, which were not included in the reference population (~ 0.27 for FY, 0.18 for MY and 0.09 for PY; Table 6). Results from linear combinations were consistent with previous BayesR analyses of traits in their original form, which were higher than obtained in a previous univariate analysis using GBLUP [see Additional file 7: Table S5]. The BayesMV analysis tended to have similar or slightly higher prediction accuracies than the BayesR analyses when validated in the Holstein set (Table 6) but similar or slightly lower accuracies for the Jersey and the Australian Reds validation sets. This could be due to fewer SNPs having non-zero effects in the BayesMV compared to the BayesR analysis and SNP effects being estimated in a reference population composed mostly of Holstein animals. Estimates of bias were similar to those from a previous analysis of the traits [1] and biases failed to show consistent differences, either when comparing bias of results for the original traits to those for the linear combinations of traits, or when comparing univariate and multivariate analyses of the linear combinations.

**Table 6:** Accuracy and bias of genomic predictions for milk production^a^ traits using different reference populations and different analysis methods and when validated in Holstein, Jersey or Australian Red animals

Analysis method^b^	Reference dataset	Validation dataset	Accuracy^c^			Bias		
			FY	MY	PY	FY	MY	PY
BayesR^d^	Holstein	Holstein	0.63	0.62	0.58	1.22	0.89	1.02
BayesR_LC	Holstein	Holstein	0.65	0.62	0.57	1.17	0.91	0.99
BayesMV	Holstein	Holstein	0.65	0.63	0.59	1.21	0.89	1.03
BayesR^d^	Hol_Jer	Holstein	0.65	0.63	0.58	1.25	0.89	0.99
BayesR_LC	Hol_Jer	Holstein	0.65	0.62	0.58	1.14	0.90	0.97
BayesMV	Hol_Jer	Holstein	0.66	0.63	0.58	1.17	0.87	0.97
BayesR^d^	Jersey	Jersey	0.56	0.70	0.72	0.89	0.98	1.24
BayesR_LC	Jersey	Jersey	0.57	0.70	0.72	0.70	1.05	1.17
BayesMV	Jersey	Jersey	0.55	0.70	0.71	0.81	1.00	1.11
BayesR^d^	Hol_Jer	Jersey	0.56	0.69	0.71	0.93	0.95	1.18
BayesR_LC	Hol_Jer	Jersey	0.58	0.69	0.73	0.92	1.00	1.20
BayesMV	Hol_Jer	Jersey	0.55	0.66	0.69	0.92	0.96	1.15
BayesR^d^	Hol_Jer	Aust Red	0.26	0.22	0.10	0.89	0.56	0.38
BayesR_LC	Hol_Jer	Aust Red	0.28	0.20	0.12	0.87	0.53	0.41
BayesMV	Hol_Jer	Aust Red	0.26	0.14	0.07	0.75	0.34	0.25

^a^Milk production traits were fat yield (FY), milk yield (MY) and protein yield (PY)

^b^Methods were either BayesR on raw phenotypes (BayesR), linear combinations of traits analyzed with univariate BayesR (BayesR_LC) or the multivariate BayesMV method

^c^Standard errors are approximately 0.062 for Holstein, 0.098 for Jersey and 0.074 for Australian Red predictions

^d^Univariate results from Kemper et al. [1]

## Discussion

Many genomic prediction and QTL mapping methods consider only one trait at a time. In this paper, we present a multivariate method for simultaneous QTL mapping, analysis of genetic architecture, and genomic prediction, i.e. BayesMV (a multivariate form of BayesR). BayesMV uses information on multiple traits for the selection of SNPs to be included in the model and within-trait information for estimation of the effects of the selected SNPs. It assumes that SNPs fall into one of two classes: either they have no effect for any trait or they have an effect for one or more traits.

The results using simulated data showed that BayesMV can have three advantages over BayesR: BayesMV correctly identifies QTL that affect multiple traits, it maps the QTL more precisely, and it predicts breeding values with greater accuracy. However, when no pleiotropic SNPs were simulated, BayesMV still identified some SNPs with effects for both simulated traits. High false-discovery rates when no pleiotropic QTL are present seems a common problem for multi-trait methods that assume pleiotropic effects [9]. The size and number of QTL that affect traits also influences the performance of multi-trait methods. For example, Jia and Jannink [17] used simulation to show that non-linear multivariate methods can outperform multivariate GBLUP in terms of genomic prediction accuracy when QTL of large effect segregate for traits. Furthermore, Chen et al. [12] showed that improvements in the accuracy of genomic predictions using a multivariate non-linear Bayesian approach were modest (compared to univariate methods) when the traits were affected by many segregating QTL compared to traits with few QTL of large effect. Experimental power to detect QTL (i.e. sample size) also influences the conclusions drawn, with non-linear methods tending to have higher accuracy for genomic predictions compared to GBLUP as sample size increases [1]. Thus, our simulation results should be interpreted with caution since they depend on how the simulations are constructed. However, they do demonstrate that BayesMV can outperform single-trait methods when the genetic architecture of the analyzed traits matches the assumptions that underlie the BayesMV model.

For the real milk yield data from dairy cattle, the advantage of BayesMV over BayesR was not as clear. For example, BayesMV showed limited advantage over BayesR in accuracy of genomic prediction, which is not entirely unexpected. With pedigree-based BLUP, multi-trait EBV are only slightly more accurate than single-trait EBV when all traits are measured on all animals and the traits have similar heritability [29]. Using genomic prediction for psychiatric disorders, Maier et al. [30] observed a 2 to 3% increase in predictive ability for schizophrenia, bipolar disorder, and major depressive disorder when using multivariate GBLUP compared to the univariate models. These data included a large number of missing records (i.e. each individual was recorded for only one trait), traits with moderate and similar heritabilities (~ 0.25), and moderate genetic correlations between traits (~ 0.4 to 0.6). These results agree with several simulations that used GBLUP multivariate genomic predictions [17, 18]. Thus, for genomic prediction, the advantage of multivariate over univariate GBLUP depends on the genetic correlation between traits and the number of (new) records contributed by the trait(s) added to the analysis. Similar conclusions have been drawn for multi-trait versus univariate GWAS models [9] and for prediction accuracies of non-linear multivariate versus univariate models [17]. That is, multivariate models increase the accuracy of genomic predictions for low heritability traits that have a strong genetic correlation with a trait that has moderate to high heritability and when additional records measured on a trait with high heritability are added to the analysis.

Studies that implement multi-trait genomic prediction methods are often motivated by multi-breed prediction problems and use the flexibility that is inherent in these models to share information across breeds [12–16]. For example, BayesRS uses location-specific priors to share information across breeds about the location of QTL regions [14], while other implementations accumulate evidence for QTL across multiple breeds and estimate SNP effects within breed [12, 13]. Thus, information that is shared in both the multi-breed approach and our approach impacts the SNP selection step, rather than the estimation step, of the analysis. Our method could easily be applied to multi-breed genomic prediction problems (with the simplification that there is no error in the covariance between traits).

Single-trait methods, such as BayesR, often identify SNPs that are close to each other but where each SNP appears to be associated with a different trait. The question then arises whether there is one QTL that affects both traits (pleiotropy) or two linked QTL that each affect one trait. The BayesMV method attempts to solve this question but its ability to do so is limited by the information contained in the data. BayesMV makes no assumptions about presence of pleiotropic (one QTL affecting more than one trait) versus linked QTL (multiple QTL each affecting one trait). Any particular SNP can have effects for none, some, or all traits. In the simulated data, BayesMV estimates the traits that each SNP was associated with moderately well but not perfectly, as shown by the results. The high degree of LD in dairy cattle populations may make it difficult to distinguish between pleiotropic QTL and multiple non-pleitropic QTL that are in high LD and each associated with one trait. Fitting all SNPs simultaneously partly accounts for LD but it is impossible to distinguish between pleiotropy and linkage if the linked QTL are in perfect LD. Thus, although our method is motivated by pleiotropic QTL, we cannot distinguish pleiotropy from tightly linked loci. The distinction, between pleiotropy and linkage, is important for QTL discovery but is inconsequential for genomic prediction.

In the real data, the five independent chains for the BayesMV implementation all showed that most SNPs either have no effect or have effects for all three traits. These results are markedly different from the single-trait BayesR results, where less than 100 SNPs had effects for more than one trait (Table 5). In spite of these apparent differences in estimated architectures, the accuracy of prediction was similar between the multi-trait and single-trait methods (Table 6). We speculate that the high LD in cattle, coupled with the traits being highly polygenic and having many more predictors than records ($p \gg n$) results in many possible solutions for prediction that yield similar accuracies. However, our results do show that one of these possible solutions includes a situation where most selected SNPs have effects for both milk volume and composition. Further information on pleiotropy versus linkage can be gained from the pattern of effects of each SNP across the three traits. For instance, the region identified on chromosome 3 appeared to harbor two close QTL, one at 15.4 Mbp affecting protein yield and one at 15.6 Mbp affecting milk yield (see Additional file 4: Table S3). In contrast, the SNPs on chromosome 14 at 1 to 2 Mbp, all had the same pattern of effects for the three traits and may all be tracking *DGAT1*. In this case, BayesMV may fit multiple SNPs for one QTL because the causal mutation is not in the data and because of the uncertainty caused by limited sample size.

We analyzed uncorrelated linear combinations of the three traits to reduce computing time and to minimize pleiotropy. Since milk, fat and protein yields are highly genetically correlated, the presence of pleiotropic QTL is highly plausible. Therefore, an analysis based on the three original traits was certain to find extensive pleiotropy. The transformation that was applied (based on the residual covariance matrix) does not guarantee zero genetic correlations but in the case of milk, fat and protein yields, the genetic correlations of the transformed traits were assumed low. However, a general procedure would be a canonical transformation, e.g. [31], where variables are both environmentally and genetically uncorrelated.

A consistent finding for both the BayesMV and the univariate BayesR analyses was that 3000 to 4000 SNPs have non-zero effects for milk yield traits. The BayesMV analysis may imply that most of the QTL tracked by these SNPs affect all three traits. The GWAS of detailed milk composition supported this conclusion by showing that, in some cases at least, the same SNPs were associated with additional milk composition traits, such as the concentration in milk of beta-lactoglobulin and potassium.

The major difference that we observed between the BayesMV and BayesR methods was that BayesMV identifies SNPs with effects for all traits in the analysis. Using the linear combinations of traits, BayesMV identified a subset of SNPs that adequately explained variation in multiple traits. This has potential practical value because it means that we could identify a limited number of SNPs that could be genotyped rather than imputed and these SNPs could be used for multiple-trait EBV calculation. However, LD between the causal mutations and SNPs may differ between breeds, which may limit the realized advantage of BayesMV in multi-breed data (compared to univariate methods), particularly when relying on high-density SNPs. The application of BayesMV to sequence data could overcome this limitation and should increase power to identify the causal variants over univariate methods. If many unrelated traits are combined in an analysis, it is possible that a very large number of sequence variants (~ 100 K [32]) will have estimated effects for at least one trait, but the number of associated SNPs should still be much smaller than the total number of polymorphisms in the genome. This would especially be the case if the hypothesis of universal pleiotropy holds, i.e. a mutation at any locus has the potential to affect many (or most) traits [33, 34]. Simultaneously analyzing traits within a physiological domain, as we have done here, is a practical first-step to assessing and using pleiotropy.

## Conclusions

We have implemented a multivariate version of the BayesR methodology that is designed to exploit pleiotropic effects of causal loci to improve mapping ability and, in turn, improve accuracy of genomic predictions. A key feature of our method is that across-trait information is used in the selection of SNPs but effects of SNPs are estimated independently for each trait. Our model performed well in simulated datasets where causal mutations were included in the analyzed SNPs and the QTL had relatively large effects (1% of phenotypic variance). In real data, the multivariate model identified most selected SNPs to be associated with all three milk yield traits (fat, milk and protein yield) but we found little evidence to support our hypothesis that multi-trait information would improve genomic prediction accuracies in milk yield traits for dairy cattle. An advantage of the new method is that it selects a small subset of SNPs that could be used for genomic prediction for multiple traits.

### Additional files

**Additional file 1: Table S1.** Phenotypic and genetic correlation between milk yield traits, with trait heritability from the pedigree-based multi-trait model.

**Additional file 2: Figure S1.** Mean power and false-discovery rate for QTL discovery in simulated data for a single trait.

**Additional file 3: Table S2.** Posterior mean number of SNPs in each distribution [0, 0.0001, 0.001 or 0.01 of the pedigree estimated genetic variance], data from Kemper et al. [1].

**Additional file 4: Table S3.** Top 100 SNPs with the highest mean posterior probability (PP) for inclusion in the model from the Holstein/Jersey reference population using BayesMV [39–41].

**Additional file 5: Table S4.** This file contains highly significant SNPs (P < 1 × 10^−6^) from the mixed model analysis of the milk minerals and proteins.

**Additional file 6: Figure S2.** Mean posterior probability for BayesMV and BayesR, and the −log_10_(P) association test statistic between SNP and potassium concentration on bovine chromosome 19 near *KCNJ2.*

**Additional file 7: Table S5.** Genomic prediction accuracy and bias from the univariate GBLUP model, data from Kemper et al. [1].

**Additional file 8: Figure S3.** Genetic relationship between nine dairy and beef cattle breeds.

## Appendix 1

Wright’s fixation index (*F*_ST_) and a per-breed measure of inbreeding [2*F*_ST_/(1 + *F*_ST_)] were calculated [35] for all pair-wise combinations of the three dairy breeds (Holstein, Jersey, Australian Reds) and six additional beef breeds (Angus, Charolais, Hereford, Limousin, Murray Grey and Shorthorn, from [36]) using 610,123 autosomal SNPs. Beef breeds were included to provide additional context for the genetic relationships between the dairy breeds. The ‘ape’ package in R [37, 38] was used to construct an unrooted tree of the relationships between the breeds using the neighbor-joining method. The resultant tree [see Additional file 8: Figure S3] indicates that Jerseys are highly differentiated from all other breeds (e.g. *F*_ST_: Holstein-Jersey = 0.08; *F*_ST_: AustRed-Jersey = 0.072), including the beef breeds, and that Australian Reds are closely related to Holsteins (*F*_ST_: AustRed-Holstein = 0.033).

## Appendix 2

The model fitted to each trait is:

$${\mathbf{y}} = {\mathbf{Xb}} + {\mathbf{Za}} + {\mathbf{Wv}} + {\mathbf{e}},$$

where

${\mathbf{y}}$ is a vector of $n$ phenotypes for cows or bulls,

${\mathbf{b}}$ is a vector containing the overall mean, breed and sex effects,

${\mathbf{a}}$ is a vector of $u$ polygenic breeding values, distributed as $N(0, {\mathbf{A}}\sigma_{a}^{2}$),

${\mathbf{v}}$ is a vector of $m$ SNP effects,

${\mathbf{e}}$ is a vector of *n* residual errors, distributed as $N\left( {0,{\mathbf{R}}} \right)$,

${\mathbf{X}}$ is a design matrix allocating phenotypes to mean and sex effects,

${\mathbf{Z}}$ is a design matrix allocating phenotypes to polygenic breeding values (${\mathbf{Z}} = n$ by $u$ matrix),

${\mathbf{W}}$ is a standardized genotype matrix (${\mathbf{W}} = n$ by $m$ matrix), where $w$ is the first column of ${\mathbf{W}}$ and $w = \left( {W_{l}^{*} - \bar{W}_{l}^{*} } \right)/\sigma_{l}$, $\sigma_{l}^{2} = 2c\left( {1 - c} \right)$, $c =$ $\bar{W}_{l}^{*} /2$ and $W_{l}^{*}$ is the first column of a $n \times m$ matrix of genotype calls,

${\mathbf{A}}$ is a numerator relationship matrix,

${\mathbf{R}}$ is an error covariance matrix, where ${\mathbf{R}} = {\mathbf{E}}\sigma_{e}^{2}$ and ${\mathbf{E}}^{ - 1}$ is a $n$ by $n$ diagonal matrix of error weights. Calculation of the weights follows [1, 20], we used $\frac{{d\left( {1 - r^{2} } \right)}}{{4 - h^{2} }}$ for bulls and $\frac{{t\left( {1 - h^{2} } \right)}}{{1 + \left( {t - 1} \right)r - th^{2} }}$ for the cows (where $t$ is the number of records per cow, $r$ is the trait repeatability, $d$ is the number of daughters per sire and $h^{2}$ is the heritability of the trait).

$\sigma_{a}^{2}$ is the additive genetic variance not explained by the SNPs.

$\sigma_{e}^{2}$ is the error variance.

It is assumed that the residuals (${\mathbf{e}}$) and polygenic effects (${\mathbf{a}}$) are independent between traits. The only connection between traits is the model for the SNP effects (${\mathbf{v}}$) as explained below.

### Priors

The mean, breed and sex effects (${\mathbf{b}}$), and polygenic effects (${\mathbf{a}}$) were assigned an uninformative normal prior distribution. The prior for the variance parameters ($\sigma_{a}^{2}$ and $\sigma_{e}^{2}$) were scaled inverse Chi squared distribution with $n - 2$ degrees of freedom.

BayesMV introduces (compared to the univariate equivalent BayesR) a parameter $\varvec{ }p$, which is the proportion of unassociated SNPs (i.e. SNPs with zero effect on all traits). For SNPs in the associated class ($1 - p$), the prior distribution for the SNP effects (${\mathbf{v}}$) follows Erbe et al. [22] and assumes SNP effects are from a mixture of four zero mean normal distributions with 0, 0.0001 $\upsigma_{{\varvec{a}^{\varvec{*}} }}^{2}$, 0.001 $\upsigma_{{\varvec{a}^{\varvec{*}} }}^{2}$ or 0.01 $\upsigma_{{\varvec{a}^{\varvec{*}} }}^{2}$ variance (where $\upsigma_{{\varvec{a}^{\varvec{*}} }}^{2}$ is the genetic variance, as estimated from pedigree analysis) and mixing proportions $q_{k,j}$ for distribution *k* from trait *j*. The genetic variance was assumed known and determined prior to the analysis, i.e. see Table 4 for heritability estimates. The mixing proportions ($p$ and $q$) follow Dirichlet distributions with one pseudo count in each category. Note that the distribution from which the effect of an associated SNP is drawn for one trait is independent of that for other traits.

### Gibbs sampling

Note that in the following, the current estimates of the parameters in the Gibbs sampler (e.g. $\tilde{b}$) are distinguished from the final estimates (e.g. $\hat{b}$) using superscripts.

For each trait,

1. Sample $\sigma_{e}^{2}$ from a scaled inverse Chi squared distribution with mean equal to $\tilde{e}^{{\prime }} E^{ - 1} \tilde{e}$ and $n - 2$ degrees of freedom, where $\tilde{e} = y - X\tilde{b} - Z\tilde{a} - W\tilde{v}$, and $\tilde{b}$, $\tilde{a}$ and $\tilde{v}$ are the current estimates of the parameters.
2. Sample estimates for the mean and sex effects from a normal distribution with mean $\left[ {X^{{\prime }} R^{ - 1} X} \right]^{ - 1} X^{{\prime }} R^{ - 1} y^{*}$ (where $y^{*}$ is the phenotype *y* corrected for the current estimates of all other terms in the model) and variance $\left[ {X^{{\prime }} R^{ - 1} X} \right]^{ - 1}$.
3. Sample polygenic effects for animal $i$ from a normal distribution with mean $\left[ {Z_{i}^{{\prime }} R_{ii}^{ - 1} Z_{i} + A_{ii}^{ - 1} \sigma_{a}^{ - 2} } \right]^{ - 1} Z_{i}^{{\prime }} R_{ii}^{ - 1} y^{*}$ (where $Z_{i}$ is the row corresponding to animal $i$ in ${\mathbf{Z}}$, and $A_{ii}^{ - 1}$ and $R_{ii}^{ - 1}$ are the $i$th diagonal elements of ${\mathbf{A}}^{ - 1}$ and ${\mathbf{R}}^{ - 1}$, respectively) and variance $\left[ {Z_{i}^{'} R_{ii}^{ - 1} Z_{i} + A_{ii}^{ - 1} \sigma_{a}^{ - 2} } \right]^{ - 1}$.
4. Sample the polygenic variance from a scaled inverse Chi squared distribution with mean $\tilde{a}^{{\prime }} A^{ - 1} \tilde{a}$ and $n - 2$ degrees of freedom.

Combining the data for all traits,

- Sample the effect of each SNP as follows:
- Sample the SNPs as unassociated with probability $\frac{{p\mathop \prod \nolimits_{J} L_{j,1} }}{{p\mathop \prod \nolimits_{J} L_{j,1} + \left( {1 - p} \right)\mathop \prod \nolimits_{J} \left( {\mathop \sum \nolimits_{K} L_{j,k} q_{j,k} } \right)}}$, where $L_{j,k}$ is the likelihood of the SNP being sampled from distribution $k$ for trait $j$, $K$ is the number of distributions (i.e. $K$ = 4), $J$ is the number of traits, $p$ and $q_{j,k}$ are the mixing proportions and the first distribution for each trait has zero variance. The log-likelihood is calculated as $\ln \left( {L_{k,j} } \right) = - 0.5\left[ {\ln \left( {1 + {\mathbf{w^{\prime}}}R^{ - 1} {\mathbf{w}}\sigma_{{k,{\text{j}}}}^{2} } \right) + y_{j}^{{*{\prime }}} R^{ - 1} y_{j}^{*} - y_{j}^{*} R^{ - 1} {\mathbf{w}}v_{{k,{\text{j}}}} } \right]$, where $\sigma_{{k,{\text{j}}}}^{2}$ is the variance of distribution $k$ for trait $j$, $y_{j}^{*}$ is the phenotype for trait $j$ corrected for all other current effects in the model, ${\mathbf{w}}$ is a vector of genotypes for the current SNP and $v_{k,j} = \left[ {{\mathbf{w}}^{{\prime }} {\mathbf{R}}^{ - 1} {\mathbf{w}} + \sigma_{k,j}^{ - 2} } \right]^{ - 1} \varvec{w}^{{\prime }} {\mathbf{R}}^{ - 1} y_{j}^{*}$.
- If the SNP is sampled as unassociated, the SNP effect for all traits is set to zero and the algorithm moves onto the next SNP.
- If the SNP is sampled as associated, the distribution $k$ is sampled independently for each trait $j$ with probability $\frac{{L_{j,k} q_{j,k} }}{{\mathop \sum \nolimits_{J,K} L_{j,k} q_{j,k} }}$.
- Using the sampled distributions, the effect of each SNP is sampled for each trait from $N(v_{{k,j^{{\prime }} }}, \left[ {{\mathbf{w}}^{{\prime }} {\mathbf{R}}^{ - 1} {\mathbf{w}} + \sigma_{k,j}^{2} } \right]^{ - 1}$).
- Update $p$ by sampling from a Dirichlet distribution given by $p \sim Dir\left( {\alpha + \beta_{0} } \right),$ where $\alpha^{{\prime }} = \left( {1, 1} \right)$ is the prior and $\beta_{0}$ is the current number of markers in the associated and unassociated class.

Update $q_{j,k}$ for each trait by sampling from a Dirichlet distribution given by $q_{j,k} \sim{\text{Dir}}\left( {{{\upgamma }} + \upvarepsilon_{{{\text{k}},{\text{j}}}} } \right)$, where $\upgamma^{{\prime }} = \, \left( {1,1,1,1} \right)$ is the prior and ${{\upvarepsilon }}_{{{\text{k}},{\text{j}}}}$ is the current number of markers with effects sampled from distribution *k* for trait *j*.
