# Supplementary material for: A multi-trait Bayesian method for mapping QTL and genomic prediction
Source: Genet Sel Evol. 2018 Mar 24;50:10. doi: 10.1186/s12711-018-0377-y (PMC5866527; doi:10.1186/s12711-018-0377-y)
Supplement: Supplementary file 1 — Additional file 1: Table S1. Phenotypic and genetic correlation between milk yield traits, with trait heritability from the pedigree-based multi-trait model. [file 12711_2018_377_MOESM1_ESM.pdf]

Table S1. Phenotypic (above diagonal, $\sigma^2_{P1.P2}/\sqrt{\sigma^2_{P1}\sigma^2_{P2}}$) and genetic (below diagonal, $\sigma^2_{a1.a2}/\sqrt{\sigma^2_{a1}\sigma^2_{a2}}$) correlation between traits for milk yield traits, with trait heritability (diagonal, $\sigma^2_{a1}/\sigma^2_{P1}$) from the multi-trait model [where σ^2_{a1} (or $\sigma^2_{a1.a2}$) represents the genetic variance for trait 1 (or covariance between traits 1 and 2) and similarly σ^2_{P1} ($\sigma^2_{P1.P2}$) represents the phenotypic variance (or covariance) for the traits]. Standard errors for the estimates are shown in brackets.

	FY		MY		PY	
fat yield (FY)	0.46	(0.013)	0.55	(0.006)	0.67	(0.005)
milk yield (MY)	0.32	(0.014)	0.56	(0.014)	0.88	(0.002)
protein yield (PY)	0.53	(0.012)	0.81	(0.005)	0.49	(0.013)
