# Supplementary material for: A multi-trait Bayesian method for mapping QTL and genomic prediction
Source: Genet Sel Evol. 2018 Mar 24;50:10. doi: 10.1186/s12711-018-0377-y (PMC5866527; doi:10.1186/s12711-018-0377-y)
Supplement: Supplementary file 2 — Additional file 2: Figure S1. Mean power and false-discovery rate for QTL discovery in simulated data for a single trait. [file 12711_2018_377_MOESM2_ESM.pdf]

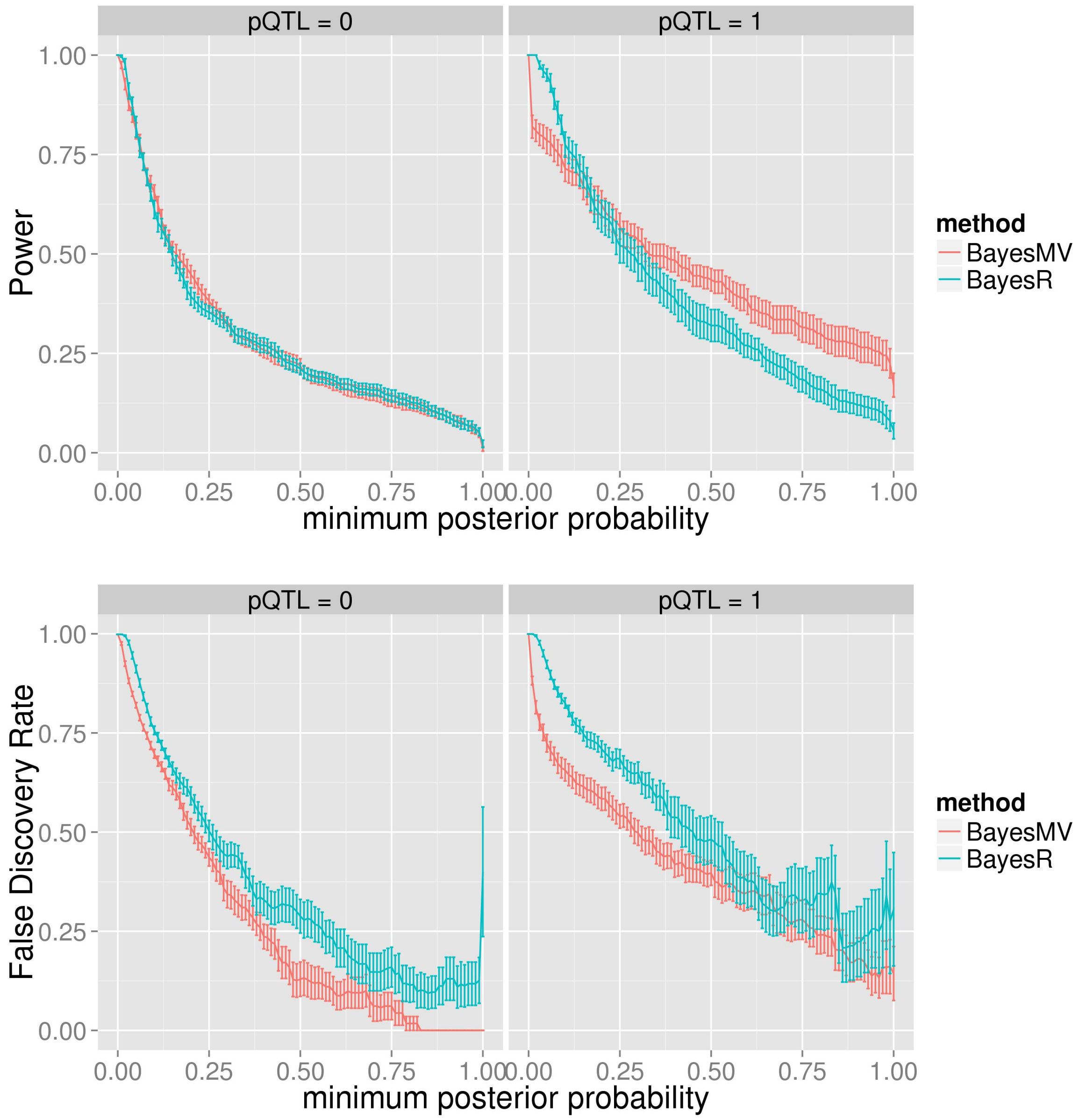

Figure S1. Mean power (top) and false-discovery rate (bottom) for QTL discovery in simulated data for a single trait. Analysis methods were either univariate (BayesR) or multi-variate (BayesMV), where there was either no pleiotropic QTL (pQTL = 0, left) or complete pleiotropy (pQTL = 1, right). Error bars indicate the standard error of the mean for 20 replicates.
