# Supplementary material for: A multi-trait Bayesian method for mapping QTL and genomic prediction
Source: Genet Sel Evol. 2018 Mar 24;50:10. doi: 10.1186/s12711-018-0377-y (PMC5866527; doi:10.1186/s12711-018-0377-y)
Supplement: Supplementary file 3 — Additional file 3: Table S2. Posterior mean number of SNPs in each distribution [0, 0.0001, 0.001 or 0.01 of the pedigree estimated genetic variance], data from Kemper et al. [1]. [file 12711_2018_377_MOESM3_ESM.pdf]

Table S2. Posterior mean number of SNP in each distribution [0, 0.0001, 0.001 or 0.01 of the pedigree estimated genetic variance ($\sigma^2_{\boldsymbol{a}^*}$)]. Data from Kemper et. al. [1].

Reference dataset	$\sigma^2_{\boldsymbol{a}^*}$	Fat yield (FY)	Milk yield (MY)	Protein yield (PY)
Hol_Jer	0	627,584	627,787	627,377
	0.0001	4389	4155	4583
	0.001	23	54	37
	0.01	6	6	5
Holstein	0	627,973	628,084	627,605
	0.0001	3968	3834	4352
	0.001	53	78	40
	0.01	7	6	5
Jersey	0	628,048	628,967	628,454
	0.0001	3897	2960	3469
	0.001	49	68	73
	0.01	8	6	6
