# Supplementary material for: A multi-trait Bayesian method for mapping QTL and genomic prediction
Source: Genet Sel Evol. 2018 Mar 24;50:10. doi: 10.1186/s12711-018-0377-y (PMC5866527; doi:10.1186/s12711-018-0377-y)
Supplement: Supplementary file 4 — Additional file 4: Table S3. Top 100 SNPs with the highest mean posterior probability (PP) for inclusion in the model from the Holstein/Jersey reference population using BayesMV [39–41]. [file 12711_2018_377_MOESM4_ESM.pdf]

Table S3. Top 100 SNP with the highest mean posterior probability (PP) for inclusion in the model from the Holstein/Jersey reference population using BayesMV. Shading indicates grouping of the QTLs according to their direction of effects on each of the traits; either fat (FY), milk (MY) or protein (PY) yield#.

SNP name	BTA	position (bp)	PProb	FY	MY	PY	Candidate gene
BovineHD0100011295	1	39389202	0.71	-	-	-	
BovineHD4100000422	1	73302604	0.47	-	-	-	
BovineHD0100025771	1	90629428	0.47	+	-	-	
BovineHD0100033516	1	118736041	0.56	(-)	-	(-)	
BovineHD0100041639	1	144440699	0.49	-	-	-	SLC37A1 [23]
BovineHD0200012097	2	41696846	0.79	+	+	+	
BovineHD0200033645	2	116733758	0.49	(+)	-	-	
BovineHD0200035007	2	120723058	0.74	+	-	-	
BovineHD0200035125	2	121128861	0.57	-	+	+	
BovineHD0200036222	2	124656921	0.56	+	-	-	
BovineHD0200037477	2	129028018	0.48	(+)	+	-	
BovineHD4100001819	3	7937124	0.58	+	-	+	
BovineHD0300004800	3	14837576	0.64	+	-	+	
BovineHD0300005092	3	15546316	1.00	-	-	+	SLC50A1 [39]
BovineHD0300005121	3	15638565	0.61	-	+	-	
BovineHD0300010269	3	32929783	0.51	+	+	+	
BovineHD0300030794	3	107112873	0.44	+	+	+	
BovineHD0300032672	3	113140396	0.63	+	-	-	
BovineHD0400003444	4	11360411	0.81	-	+	+	
BovineHD0400006694	4	22682241	0.47	+	-	-	
BovineHD0400033443	4	115414057	0.49	+	-	-	
BovineHD0500023925	5	84525667	0.60	+	+	+	
BovineHD0500025181	5	88795885	0.54	-	-	-	
BovineHD0500026635	5	93843392	0.47	+	-	-	
BovineHD0500026645	5	93875727	0.60	+	-	+	
BovineHD0500026860	5	94586978	0.80	+	-	-	MGST1 [26]
BovineHD0500034287	5	118099483	0.60	+	+	-	
BovineHD0500034328	5	118206134	0.46	+	-	+	
BovineHD0600007970	6	28759913	0.69	-	-	-	
BovineHD0600010605	6	38284780	0.46	+	-	+	ABCG2 [40]

BovineHD0600011060	6	40583958	0.73	-	+	(-)	
BovineHD0700008359	7	29679398	0.47	+	+	+	
BovineHD0700029964	7	102574897	0.45	-	-	-	
BovineHD0900025344	9	89738098	1.00	+	-	-	
BovineHD1100022618	11	78888103	0.59	-	+	+	
BovineHD1100030073	11	103317601	0.60	-	+	+	PAEP / LGB
BovineHD1200004660	12	15637232	0.59	-	-	-	
BovineHD1200009378	12	31866913	0.48	(+)	+	(+)	
BovineHD1200009963	12	33827073	0.47	-	+	+	
BovineHD1200019329	12	70293273	0.83	-	-	-	
BovineHD1200021971	12	77514314	0.48	-	+	-	
BovineHD1200022581	12	79220402	0.47	-	+	-	
BovineHD1200024310	12	83938103	0.48	-	-	-	
BovineHD1400000152	14	1439476	0.60	+	-	-	
BovineHD1400000216	14	1736599	0.80	-	+	+	
ARS-BFGL-NGS-4939	14	1801116	0.60	+	-	-	DGAT1 [19]
ARS-BFGL-NGS-18365	14	2117455	0.60	+	-	-	
BovineHD1400000325	14	2264522	0.80	-	+	+	
BovineHD1400000482	14	2940147	0.60	+	-	-	
BovineHD1400000563	14	3195864	0.51	+	-	-	
BovineHD1400014375	14	50620705	0.60	-	+	-	
ARS-BFGL-NGS-94979	14	63946731	0.45	+	-	-	
BovineHD1400018384	14	65751278	0.45	+	-	(-)	
BTB-01324160	16	1608132	0.66	-	+	-	
BovineHD1600011561	16	40910562	0.57	(-)	-	+	
BovineHD1700018934	17	65757287	0.45	-	-	-	
BovineHD1700020108	17	68863608	0.80	+	-	-	
BovineHD1800006936	18	22568721	0.60	+	-	-	
ARS-BFGL-NGS-35499	18	50727653	0.49	+	-	-	
BovineHD1800016751	18	57508328	0.50	+	-	-	
BovineHD1800017015	18	58414597	0.60	+	-	-	
BovineHD1800017349	18	60019327	0.65	+	-	-	
BovineHD1900002660	19	9439368	0.48	-	-	-	
BovineHD1900006099	19	21189263	0.60	+	-	-	
BovineHD1900010587	19	36569291	0.44	(+)	-	-	
BovineHD1900017409	19	60753539	0.45	-	+	+	

BovineHD1900017548	19	61134515	0.48	-	-	(+)	KCNJ2 (Fig. S2)
BovineHD2000001299	20	4035913	0.80	+	-	-	
BovineHD2000004455	20	14210526	0.46	-	-	-	
BovineHD2000008924	20	30616782	0.60	+	-	+	
BovineHD2000009067	20	31440760	0.50	+	-	+	
BovineHD2000009139	20	31683746	0.60	+	-	+	GHR [41]
BovineHD2000009457	20	33027949	0.53	+	-	+	
BovineHD2000011122	20	39182594	0.57	-	-	-	
BovineHD2000016265	20	58530550	0.51	+	-	+	
BovineHD2100003508	21	13486454	0.48	+	-	(+)	
BovineHD2100011339	21	39427041	0.45	+	-	+	
BovineHD2200009997	22	35026734	0.49	-	-	-	
BovineHD2200016044	22	56036171	0.54	+	-	-	
BovineHD2300008118	23	28679974	0.75	-	-	-	
BovineHD2300013060	23	45011198	0.46	+	+	(-)	
BovineHD2300013065	23	45040309	0.45	-	-	(+)	
BTB-02081535	23	52285248	0.64	-	+	+	
BovineHD2400005905	24	21838065	0.46	-	-	-	
BovineHD2400009809	24	35671115	0.54	-	+	+	
BovineHD2500010489	25	37750221	0.46	+	(+)	+	
BovineHD2600004967	26	19098039	0.48	-	+	+	
ARS-BFGL-NGS-57448	27	36155097	0.78	+	-	-	GPAT4 [25]
BovineHD2700011956	27	41283869	0.53	-	-	+	
BovineHD2700012081	27	41645455	0.55	-	-	-	
BTA-63683-no-rs	28	18663296	0.87	-	-	-	
Hapmap51965-BTA-101198	28	36314835	0.44	-	+	+	
BovineHD2800012748	28	44320344	0.55	+	+	+	
BovineHD2900010990	29	36306532	0.44	+	-	+	
ARS-BFGL-NGS-89746	29	49478288	0.60	+	-	+	
BovineHD3000002266	X	6612143	0.60	+	+	+	
BovineHD3000007194	X	22166972	0.60	+	-	-	
BovineHD3000015538	X	53100212	0.55	(+)	+	-	
ARS-BFGL-NGS-14575	X	133958503	0.48	-	+	(+)	
BovineHD3000040468	X	140263353	0.44	-	-	-	

#absolute SNP effects < 1×10^{-3} are shown in brackets.

There are 4 QTL types:

FY	MY	PY	Comment
+	+	+	Increase in milk volume, fat and protein yield
-	+	+	Decrease in fat yield, increase in milk volume and protein yield
+	-	+	Increase in milk solids (fat and protein yield) and decrease milk volume
+	+	-	Increase in milk volume and fat yield, decrease protein yield

References

39. Kemper KE, Hayes BJ, Daetwyler HD, Goddard ME. How old are QTL and how widely do they segregate? J Anim Breed Genet. 2015,.132:121-34.
40. Cohen-Zinder M, Seroussi E, Larkin DM, Loor JJ, Everts-van der Wind A, Lee JH, et al. Identification of a missense mutation in the bovine ABCG2 gene with a major effect on the QTL on chromosome 6 affecting milk yield and composition in Holstein cattle. Genome Res. 2005; 15:936-44.
41. Blott S, Kim JJ, Moisio S, Schmidt-Kuntzel A, Cornet A, Berzi P, et al. Molecular dissection of a quantitative trait locus: a phenylalanine-to-tyrosine substitution in the transmembrane domain of the bovine growth hormone receptor is associated with a major effect on milk yield and composition. Genetics. 2003;163:253-66.
