# Supplementary material for: A multi-trait Bayesian method for mapping QTL and genomic prediction
Source: Genet Sel Evol. 2018 Mar 24;50:10. doi: 10.1186/s12711-018-0377-y (PMC5866527; doi:10.1186/s12711-018-0377-y)
Supplement: Supplementary file 6 — Additional file 6: Figure S2. Mean posterior probability for BayesMV and BayesR, and the −log10(P) association test statistic between SNP and potassium concentration on bovine chromosome 19 near KCNJ2. [file 12711_2018_377_MOESM6_ESM.pdf]

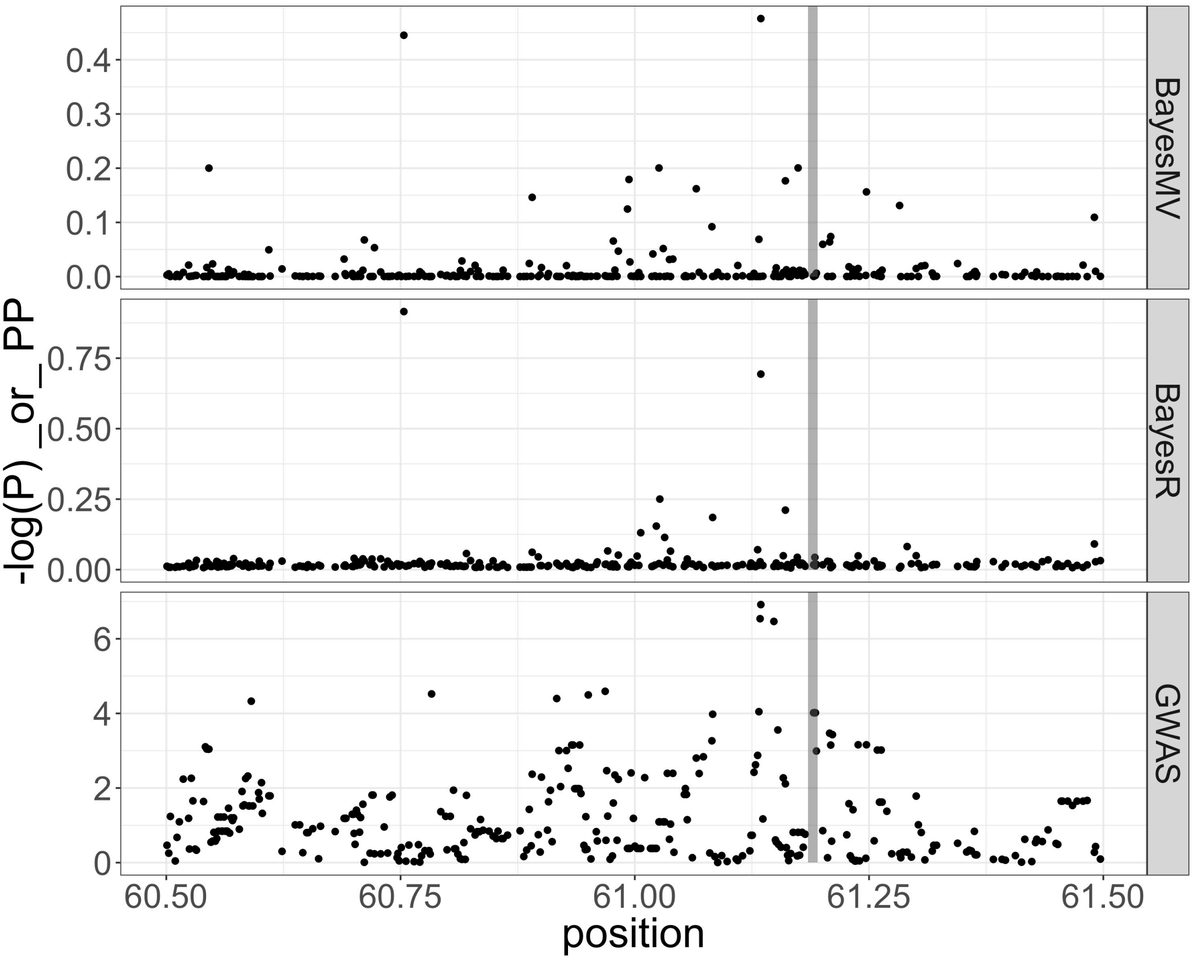

Figure S2. Mean posterior probability (PP) for BayesMV and BayesR, and the -$\log_{10}$(P) association test statistic between SNP and potassium (K) concentration on bovine chromosome 19 near the KCNJ2 gene (coding region highlighted in grey).
