# Supplementary material for: A multi-trait Bayesian method for mapping QTL and genomic prediction
Source: Genet Sel Evol. 2018 Mar 24;50:10. doi: 10.1186/s12711-018-0377-y (PMC5866527; doi:10.1186/s12711-018-0377-y)
Supplement: Supplementary file 7 — Additional file 7: Table S5. Genomic prediction accuracy and bias from the univariate GBLUP model, data from Kemper et al. [1]. [file 12711_2018_377_MOESM7_ESM.pdf]

**Table S5.** Genomic prediction accuracy and bias for the relevant reference and validation datasets from univariate GBLUP analysis, data from Kemper et al. [1].

| Reference dataset | Validation dataset | Accuracy | | | Bias | | |
|---|---|---|---|---|---|---|---|
| | | FY | MY | PY | FY | MY | PY |
| Holstein | Holstein | 0.60 | 0.58 | 0.59 | 1.18 | 0.89 | 1.06 |
| Hol_Jer | Holstein | 0.61 | 0.59 | 0.59 | 1.20 | 0.90 | 1.05 |
| Jersey | Jersey | 0.56 | 0.62 | 0.67 | 0.88 | 0.93 | 1.20 |
| Hol_Jer | Jersey | 0.58 | 0.64 | 0.69 | 0.88 | 0.91 | 1.17 |
| Hol_Jer | Aust Red | 0.17 | 0.11 | 0.04 | 0.75 | 0.32 | 0.16 |
