# Supplementary material for: A multi-trait Bayesian method for mapping QTL and genomic prediction
Source: Genet Sel Evol. 2018 Mar 24;50:10. doi: 10.1186/s12711-018-0377-y (PMC5866527; doi:10.1186/s12711-018-0377-y)
Supplement: Supplementary file 8 — Additional file 8: Figure S3. Genetic relationship between nine dairy and beef cattle breeds. [file 12711_2018_377_MOESM8_ESM.pdf]

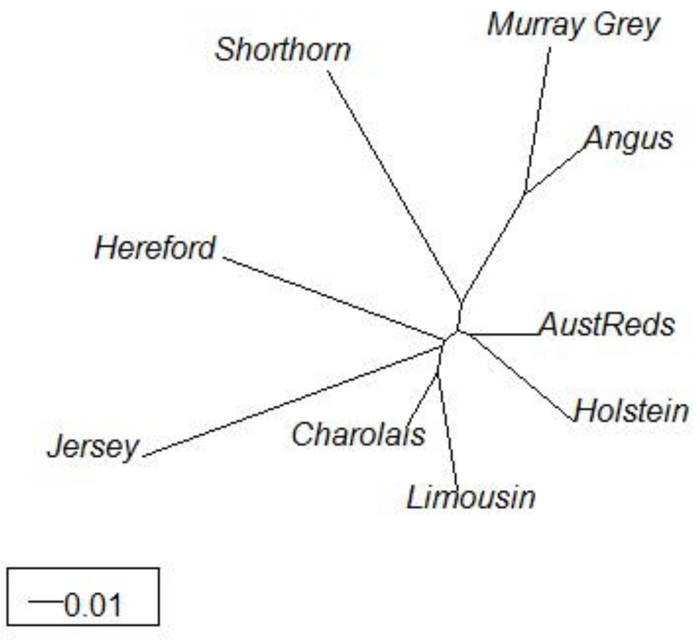

Figure S3. Genetic relationship between 9 dairy and beef cattle breeds. In current study, Holstein and Jersey dairy cattle are used in the reference population, and Australian Reds are used (exclusively) as a validation population.
